# Application of Ionic Liquids in Pot-in-Pot Reactions

**DOI:** 10.3390/molecules21030272

**Published:** 2016-02-26

**Authors:** Simge Çınar, Michael D. Schulz, Stephanie Oyola-Reynoso, David K. Bwambok, Symon M. Gathiaka, Martin Thuo

**Affiliations:** 1 Department of Materials Science & Engineering, Iowa State University, Ames, IA 50011, USA; scinar@iastate.edu (S.Ç.); so1@iastate.edu (S.O.-R.); 2 Arnold and Mabel Beckman Laboratories of Chemical Synthesis, Division of Chemistry and Chemical Engineering, California Institute of Technology, Pasadena, CA 91125, USA; mdschulz@caltech.edu; 3 Warner Babcock Institute for Green Chemistry, Wilmington, MA 01887, USA; David.Bwambok@warnerbabcock.com; 4 Skaggs School of Pharmacy and Pharmaceutical Sciences, University of California San Diego, La Jolla, CA 92093, USA; sgathiaka@mail.ucsd.edu; 5 Microelectronic Research Center, Iowa State University, 133 Applied Sciences Complex I, 1925 Scholl Road, Ames, IA 50011, USA; 6 Biopolymer and Biocomposites Research Team, Center for Bioplastics and Biocomposites, Iowa State University, 1041 Food Sciences Building, Ames, IA 50011, USA

**Keywords:** pot-in-pot reactions, unidirectional flux, cascade reactions, membrane separation, multi-step synthesis

## Abstract

Pot-in-pot reactions are designed such that two reaction media (solvents, catalysts and reagents) are isolated from each other by a polymeric membrane similar to *matryoshka* dolls (Russian nesting dolls). The first reaction is allowed to progress to completion before triggering the second reaction in which all necessary solvents, reactants, or catalysts are placed except for the starting reagent for the target reaction. With the appropriate trigger, in most cases unidirectional flux, the product of the first reaction is introduced to the second medium allowing a second transformation in the same glass reaction pot—albeit separated by a polymeric membrane. The basis of these reaction systems is the controlled selective flux of one reagent over the other components of the first reaction while maintaining steady-state catalyst concentration in the first “pot”. The use of ionic liquids as tools to control chemical potential across the polymeric membranes making the first pot is discussed based on standard diffusion models—Fickian and Payne’s models. Besides chemical potential, use of ionic liquids as delivery agent for a small amount of a solvent that slightly swells the polymeric membrane, hence increasing flux, is highlighted. This review highlights the critical role ionic liquids play in site-isolation of multiple catalyzed reactions in a standard pot-in-pot reaction.

## 1. Introduction

The idea of using membranes for reaction compartmentalization, or selective flux, is widely applied in nature, either for biosynthesis or matter transport. Membranes of all sorts have found use in nature (e.g., cell membranes), in industry and in site-isolated reactions [1]. Some of the main hindrances to efficient adoption of polymeric membranes in organic synthesis include: (i) lack of an adequate means to perturb chemical potential across the membranes; (ii) lack of polymeric or composite materials of high specific strength to withstand the mechanical and chemical stress associated with chemical reactions; (iii) challenges inherent in understanding the multi-disciplinary nature of uni-directional flux that allows one to effectively and predictably engineer chemical potential across membrane interfaces. Although the adoption of membranes in separation is well documented [1,2,3,4,5,6], especially in pervaporation [3,6,7,8,9,10,11], there is an alarming slow development in their adoption for efficient organic synthesis systems even though the need for better approaches is dire.

To understand, or develop a general platform for efficient membrane separation, three key parameters need to be considered: (i) the partition coefficient (*J_i_*) can be heavily affected by the chemical nature of the molecules to be separated and the polymeric membrane in use. By taking two miscible small molecules varying in size, polarity, and dominant moieties, one can effectively alter partition into a membrane; (ii) thickness of polymeric membrane (*L*)—hence the need for high specific strength materials that can withstand significant stress from the weight of the materials being separated; (iii) chemical potential (μ*_i_*), which in diffusing molecules will change over time but requires that other components be kept constant. Relating the activity coefficient γ_i_ to Flory-Huggins interaction parameter, χ,—from re-expression of the free energy of mixing, ΔG_m_, one can engineer partition coefficients across a polymeric thin film through felicitous choice of solutions across the interface, and hence, achieve unidirectional flux. The use of thin polymeric membranes in conjunction with ionic liquids co-solvents in pot-in-pot reactions exemplifies the application of the three parameters listed above to engineer unidirectional flux across thin polymeric membranes. This mini-review focuses on: (i) demonstration of the use of ionic liquids, organic salts that are liquids at or below room temperature, in pot-in-pot reactions; and (ii) emphasizes how empirical control of partition coefficients across polymeric interfaces can allow one to realize systems that are otherwise impossible. To help achieve this, an introduction into the unique properties of ionic liquids and a review of fundamental diffusion and flux laws is given, followed by demonstration of the application of these laws and properties in design of simple but efficient chemical reactions systems that allow multiple reactions to be performed in polymer-in-glass pots in the so-called pot-in-pot reactions. A prelude to these reactions is the entrapment of a catalyst in a polymeric pot (thimble) that serves as a partition barrier where only the reagents, or reaction products, can diffuse into the polymer-catalyst composite (from partial diffusion of the catalyst into the polymer membrane) and the product of a chemical transformation diffuses through. Success in these partition-controlled, functional group selective reactions allow for the design of systems that can isolate incompatible reaction systems from each other while effecting multi-step transformation of a feed reagent.

### Unique Properties of Ionic Liquids

**Figure 1:**
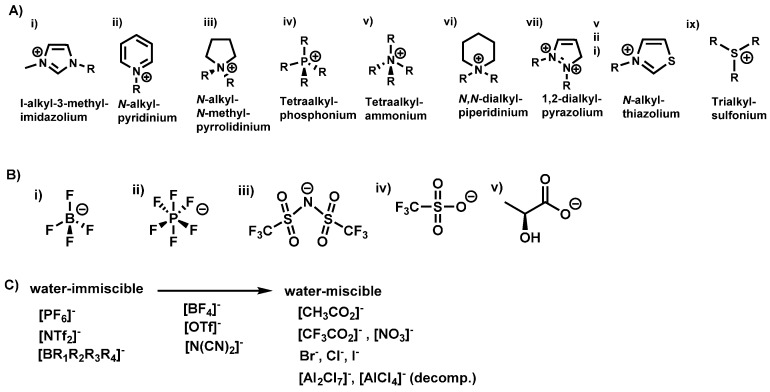
Representative common cations (**A**) and anions (**B**) used in the synthesis of ILs. Water miscibility of various anions used to synthesize ILs (**C**).

Interest in ionic liquids (ILs) continues to grow beyond academic research to industrial applications due to their unique properties as designer materials. In recent years, their use as solvents in chemical reactions has seen a significant increase due to their documented utility in organic synthesis [12,13,14] and catalysis [15,16]. ILs have received considerable attention as non-volatile, recyclable solvents in organic synthesis and catalysis [17,18,19,20]. For example, they have been used as green, environmentally benign and recyclable solvents for palladium-catalyzed coupling reactions [21], desulfurization of liquid fuels [19], denitrification [20], dissolution of pharmaceutical drugs [18], and cellulose processing [17]. Ionic liquids are defined as organic salts with melting point at or below 100 °C [22,23,24]. The upper temperature limit was used to distinguish ionic liquids as low melting salts compared to inorganic salts that have significantly higher melting points [22,23,24]. Thus, at room temperature, ionic liquids can be liquids (room temperature ionic liquids) [25,26] or solids (frozen ionic liquids) [22,23,24]. These solvents are usually composed of a low symmetry organic cation and a weakly coordinating inorganic or organic anion with a diffuse negative charge (examples in Figure 1) [23,25,26]. The low melting point of ionic liquids is due to asymmetry between the cation and anion resulting in poor crystal packing between these constituent ions [15,16].

Compared to only about 600 molecular solvents currently used, approximately 10^18^ ILs can be generated from possible combinations of various cations and anions [27]. As a result, ionic liquids are considered designer solvents because properties like; solubility, polarity, melting point, viscosity, and density, can be tailored to meet specific applications by varying the cation or anion [22,24,28]. Various studies have worked on understanding the structure-property relationship of these solvents in order to elucidate the dominant forces that impact their properties, enabling them to influence chemical reactivity. For instance, studies on the geometric and energetic properties on imidazolium cation-containing ILs have revealed that the anion prefers to reside over the imidazole ring forming a hydrogen bond with the acidic C_2_-H group [28]. Other non-covalent interactions like coulombic forces [29], van der Waals interactions [27] and π-π stacking [25] have been found to be key and dominate the behavior of ILs hence significantly influence their properties.

Ionic liquids have unique properties including negligible vapor pressure, non-flammability, high thermal stability, high conductivity, high solubility for various compounds (polar/non-polar, organic/inorganic) and recyclability [29]. The low vapor pressure and high thermal stability of ionic liquids is due to large cohesive energy density from coulombic interactions between the constituent ions [21]. These attractive properties of ILs enable their use as green, non-volatile, non-flammable, recyclable and thus safer, environmentally benign solvents to replace volatile, flammable, toxic organic solvents in synthesis.

## 2. Ionic Liquids in Pot-in-Pot Reactions

**Figure 2:**
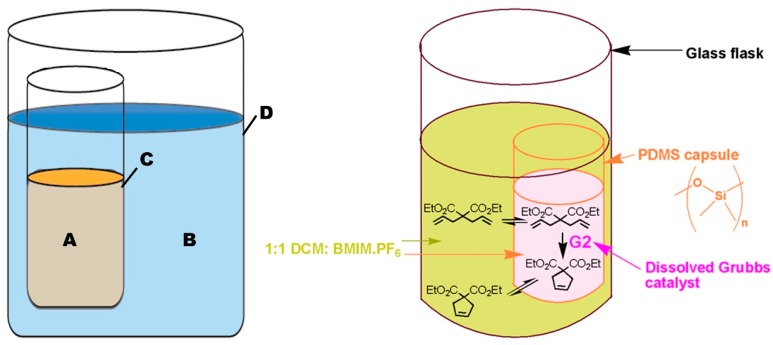
Schematic representation of a pot-in-pot reaction set-up. The reaction in the first pot is performed in a mixture of ionic liquids and a medium polarity solvent (**A**) and separated from a second reaction medium by a thin polymeric membrane (**C**). A second reaction medium (**B**) is placed outside the first pot (**C**) and is used to transform the product of the first reaction into a new product. The large reaction pot (**D**) is normally a conventional glass reaction flask. Medium (**A**) contains ionic liquids while (**B**) does not; allowing unidirectional flux. An example of the reaction process in absence of unidirectional flux is shown [31].

“Pot-in-pot reactions” refers to a synthetic approach involving site-isolation of catalysts and/or reagent(s) using a semi-permeable membrane (inner pot) inside a second impermeable flask (outer pot) (Figure 2) [30]. Products formed in the inner pot can flux through the semipermeable membrane and are collected in the outer pot; either for further reaction or for isolation. Fresh reagents can be added into pot 1, and the process is repeated for multiple cycles. Polydimethylsiloxane (PDMS) membrane is commonly used for pot-in-pot reactions because it can readily be made into various shapes and sizes and affords good flux selectivity for large or polar molecules and high flux rates for small or medium polarity organic molecules [31].

There are several studies aimed at controlling transport of ionic liquids across polymeric membranes. Engineering the structure of the ionic liquid is an obvious starting point, or their incorporation in so called Supported Ionic Liquid Membranes (SILMs)—a detailed review of which has been offered elsewhere [32,33,34]. While these modifications are a significant advance in the application of ionic liquids, we limit our discussion to the use of simple widely available and/or easy to synthesize ionic liquid systems for liquid-liquid based unidirectional flux systems.

The success of pot-in-pot reactions in obtaining high yields of the desired products from cascade reactions depends on efficient site-isolation of; (i) a reagent from a reagent; (ii) catalyst from a reagent, (iii) catalyst from a catalyst; and/or (iv) recycling a catalyst over many reaction cycles. The great potential of ILs for efficient site isolation of the catalyst in pot-in-pot reactions is due to high solubility of organometallic catalysts in ILs and the possibility of recycling the catalyst, which is an additional environmental and economic benefit. Additionally, ILs do not poison the catalyst and do not flux through the PDMS membrane providing the required flux selectivity and potential for catalyst recycling. Details and examples on the use of ionic liquids as co-solvents to generate desired flux through the membrane and to reduce the amounts of volatile, toxic organic solvents used in some pot-in-pot reactions are described later, but first a review of relevant mass transport theories is presented.

## 3. Diffusion and Flux in Polymeric Membranes

### 3.1. Theories on Diffusion and Flux

Transport of molecular species through membranes is best understood through uni- and multi-direction diffusion theories and related laws. Diffusion, the stochastic movement of molecules, in polymer membranes is well studied and general rules have been established. Consequently, selective and/or controlled flux (the unidirectional movement/diffusion over a given thickness of material with time) has been envisioned as essential in membrane-based separation and/or purification, and forms the basis of the pot-in-pot reaction systems. To fully appreciate and extend molecular-level engineering of these reaction systems, a review of the guiding diffusion laws is necessary. Fick’s first law of diffusion relates the flux (*J_i_*), the rate of mass flow of a substance *i* per unit area, to the concentration gradient through a diffusion coefficient (*D_i_*) for dilute ideal solution or mixtures at steady state for the diffusion in the direction of x [35]. For dilute solutions, the diffusion arises from solute-solvent interactions, and solute-solute interactions are neglected. In the concentrated systems or the systems of solute with strong interactions such as ionic liquids, on the other hand, the system is far from being ideal, thus the driving force for diffusion is represented by the gradient of chemical potentials ($\partial \mu_{i}/\partial x$) rather than the concentration gradient of the corresponding substance to account for the solute-solute interactions. In the non-ideal case therefore, Fick’s first law relates flux, *J*, to the chemical potential (Equation (1)):

(1) $$J_{i}=-\frac{D_{i}C_{i}}{RT}\frac{\partial \mu_{i}}{\partial x}$$

where *C_i_* is the concentration of the species *i* and R and T are the universal gas constant and temperature, respectively.

On the other hand, in concentrated solutions, deviation of chemical potential due to solute-solute interactions can be expressed in terms of activity coefficient ($\gamma_{i}$) and mole fraction $\left(x_{i}\right)$ [36]. In concentrated solutions, therefore, flux can be related to the mole fraction and activity coefficient (Equation (2)). Despite this extension in the theories, incorporation of activity coefficient comes with the challenge that there are many solutes without a known activity coefficient. With advances in the ease with which organic compounds are made, a rapid increase in solutes has been witnessed and as such determination of the physical properties of these compounds has significantly lagged behind. Even in the cases where empirical data exists, there are significant differences between the experiments and data derived from Fick’s law, and as such, these laws can only be used as general guidelines and not as absolutes [37]:

(2) $$J_{i}=-D_{app}C\frac{\partial x_{i}}{\partial x}$$

where $D_{app}=D_{i}\left(1+d\;In\gamma_{i}/d\;Inx_{i}\right)$.

Another approach to understanding flux through polymeric membranes is by assuming that the materials are porous (that is, they have “holes” through which molecules can move) and applying diffusion theories. The molecular basis of these theories is that diffusion occurs through random walk of the molecules through the porous material, and, the molecule is considered as a spherical ball whose drag is calculated at a low Reynolds number. In a system that the solute is macromolecular rather than a very small spherical particle, diffusion also depends on the solute size and the noncovalent (secondary) interactions with the solvent. In general, the diffusion coefficient is expressed as a function of temperature, fluid viscosity, and, the solute size as indicated in the Einstein–Stokes equation (Equation (3)), which is applicable for low Reynolds number [38]:

(3) $$D=\frac{k_{B}\;T}{6\pi \eta r}$$

Here $k_{B}$ is the Boltzmann constant, η is the viscosity of the solvent, and, *r* is the radius of a spherical particle. According to this relation, larger molecules, for example, move slower or stronger secondary interactions between solute and solvent increases drag, thus impedes diffusion.

One strategy to minimize the effect of secondary interactions in pot-in-pot reaction systems is to increase the inter-chain distances in polymer membranes by reducing the degree of cross-linking, or solvating the polymer chains hence, masking their interactions with the diffusant, which also leads to swelling due to the favorable secondary interactions between the polymer and the introduced solvent. Since the adoption of pure solvent would significantly affect the chemical potential across the membrane, co-solvents are often used with the understanding that upon contact with the polymer, the polymer solvating component of the co-solvent would be intercalated between polymer chains, hence restoring the desired chemical potential, with respect to the precious catalyst, across the membrane.

A more widely used theory in diffusion/flux involves the need to understand, and hence minimize, drag. Because of the assumption that diffusion is a random walk that is heavily influenced by the viscosity of the solvent, hence secondary interactions, increasing Brownian motion through heat is often envisioned as a good strategy for increasing unimolecular diffusion. Based on the nature of secondary interactions between several molecules, thermal ramping can be used to kinetically separate molecules based on their diffusion constants. The temperature dependence of diffusion coefficient is first order and follows an Arrhenius-type relation (Equation (4)), hence the increased role of temperature in controlled uni-directional flux as depicted in the pot-in-pot reactions:

(4) $$D=D_{0}e^{-E/RT}$$

### 3.2. General Transport Across Membranes

Membranes function to control transport, and hence separation, by simultaneously introducing; (i) two interfaces, hence, partition coefficients (ϕ) that can be engineered to play the role of “gate-keepers” allowing some molecules to penetrate the membranes while keeping the solvents at bay; (ii) selectivity as the molecules diffuse across the membrane due to differences in diffusion constants; and (iii) modifying chemical potential, in and outside the membrane, due to changes in concentration gradients as diffusant partition into, and out of, the membranes. Permeability (P), the ability of a material to allow molecules to flux through them, is not an intrinsic property, but is the product of solute partition coefficient (ϕ) and the diffusion coefficient ($D_{m}$) per unit of membrane thickness (L) (Equation (5)). This expression of permeability, therefore, implies that selective flux is inversely dependent on the thickness of the membrane used, and as such thin membranes are desired for effective pot-in-pot reactions. This qualification, however, is compounded by the need for mechanical strength, which in the case of polymers is dependent on strong inter-chain interactions or, for thermosets, on the strength and/or degree of crosslinking. This is a materials design paradox that has been overcome by running reactions in asymmetric volume quantities across the first pot.

(5) $$P=\frac{D_{m}\phi }{L}$$

As mentioned above, for characterization of the transport properties, diffusion coefficient, which depends on the concentration of the solute on the surface of a membrane, should be known. For a system where the concentration changes on the surfaces occur much slower than the diffusion process, then the system could to be assumed to be in a quasi-steady state and, as such, Fick’s law (Equation (2)) can be applied [39]. In other words, the time required for the solute to diffuse across the membrane could be assumed much shorter than the time required for the solute concentration to change on either side of the membrane.

**Figure 3:**
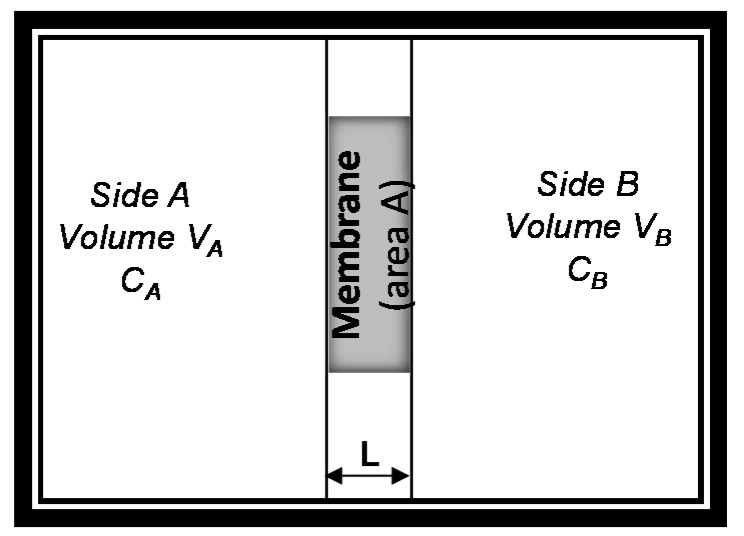
Schematic for solution of Fick’s first law of diffusion for transport through membranes.

Integrating Fick’s laws, and associated boundary limits, with the discussion on role of secondary interactions and membrane thickness, one can envision a solution to these equations that can be understood as schematically and mathematically shown (Figure 3 and Equation (6)). Assuming that the initial concentration of solute in compartment A, $C_{A}$, is equal to $C_{0}$ while the one in compartment B is zero (Figure 3), then the solution becomes [39]:

(6) $$In\left(\frac{2C_{A}-C_{0}}{C_{0}}\right)=-\frac{2A_{m}D_{m}\phi t}{VL}$$

### 3.3. Role of Ionic Liquids in Selective Uni-Directional Flux in Polymer Membranes

The use of ionic liquids, with their highly ionic nature—hence limited partition into most polymers, is desirable in attaining a quasi-steady state. This allows for the above relations to be deployed in the use of polymeric membranes as tools in the partition of different organic molecules across two diametrically opposite mediums. By initiating the flux process with the ionic liquid as the solvent for the diffusant, little to no change would be observed in terms of global ionic liquid composition, hence no significant change in the chemical potential. When the minor component partitions into the membrane, adopting Payne’s model of diffusion (discussed below), then we can infer that favorable enthalpic interactions will drive the diffusant across the membrane, through a series of favorable secondary interactions, while the relative concentration of an organometallic catalyst is held constant due to higher solubility in the ionic liquid relative to the polymer.

Payne and Wayne [10] reported that it is the solubility of the diffusant in the membrane, rather than the diffusion coefficient or the permeant size that strongly determines the permeation of organics through polymer membrane. They define the diffusion process as a three dimensional dynamic adsorption process in which permeant moves from one adsorption site to another during its transport. The strength of permeant—membrane interactions determine the dwell time, that is, the time spent on each site by an adsorbed molecule. The diffusion coefficient (D), then, can be defined in terms of dwell time (τ), the jump length (λ), activation energy characterizing the permeant—polymer physi-sorption bond (E), temperature (T) and gas constant (R) as given in Equation (7):

(7) $$D=\frac{\lambda^{2}}{2\tau_{0}}\text{exp}\left(-\frac{E}{RT}\right)$$

With the assumption of Langmuir type adsorbed layer that effectively covers, hence neutralizes, the permeant—membrane interaction sites through the membrane volume, the concentration dependent diffusion coefficient (D_c_) can be expressed as shown below(Equation (8)), where $C_{m}/C_{s}$ is the ratio of the mobile permeant concentration to the adsorption site concentration, and $\tau_{s}/\tau_{t}$ is the ratio of the adsorption dwell time to the transit time between sites: [5]

(8) $$D_{c}=\frac{{\left(1+\frac{C_{m}}{C_{s}}\frac{\tau_{s}}{\tau_{t}}\right)}^{2}D}{{\left(1+\frac{C_{m}}{C_{s}}\frac{\tau_{s}}{\tau_{t}}\right)}^{2}+\frac{\tau_{s}}{\tau_{t}}}$$

It is therefore important, from Fickian and Payne’s diffusion models that as the concentration of the diffusant (reaction product) changes, other components of the reaction mixture remains constant hence the need for a solvent that does not partition into the polymer and biases partitioning of the catalyst towards the reaction medium. Depletion of the solvent and/or the catalyst (either by diffusion into the membrane, decomposition, or precipitation) will make it challenging to recycle the catalysts (a key advantage in using membranes) or have continuously active catalysts over prolonged reaction times.

In summary, in pot-in-pot reactions, flux can be tuned by felicitous choice of solvents, that is, either: (i) engineering the solubility of the reactants and products in the reaction medium in the first or second pot, and relating this to solubility in the polymer matrix; (ii) engineering the partition coefficients across the polymer-solvent interfaces to maximize selective permeation into the polymer. One approach to achieve this is to use solvents in which the reactants and products are more soluble on the inside pot, hence use a small volume in the inner pot, and use one where solubility of the reaction product is lower on the outside pot, hence the need for a larger volume to drive the flux.

According to the transport equations presented in the previous section, selective transport can be enhanced by tuning several parameters. Increasing the amount of solvent in the second pot, for example, will decrease the concentration of solute in that pot, and thus enhance transport by increasing the concentration gradient between pots. Based on the effect of diffusion path on flux, thinner membranes are desired, but even with these, the solute needs to remain dissolved at all times. Ionic liquids do not partition into PDMS and as such they are ideal candidates for this purpose. As the strength of the membrane is vital to contain the reaction mixture, trade-offs between the mechanical properties of the membrane and its thickness should be considered carefully. The use of ionic liquids, with their low-density, compared to halogenated analogs for comparable polarity, ensures that the effect of mechanical stress on the membrane is minimized. In the section below, several examples of these reactions are discussed to highlight how ionic liquids have played an important role in catalytic pot-in-pot reaction systems. In the absence of ionic liquids, the use of organometallic reagents becomes significantly challenging in part due to decomposition, precipitation, and in some cases, diffusion across the membranes.

## 4. Examples of Pot-in-pot Reactions

Molecular transformations are almost always performed sequentially, often with a purification procedure between reactions. The necessity of this separation arises from the fact that most reaction conditions are mutually incompatible. While “temporal isolation” of individual reaction steps is probably the most common approach to circumvent reaction incompatibility, site-isolation is almost always more efficient, particularly because it avoids intermittent purification steps [40,41]. Chemical transformations, however, involve many components (e.g., catalysts, reagents, solvents, reactants, *etc.*), and approaches to site-isolation focus only on one of these elements (mounting catalysts on solid supports, for example) [42]. In order to such approaches to be successful, all of the other components must be compatible with the reaction cascade, while ensuring that the target reactions occur only in the desired order and on the desired substrates. Additionally, many approaches to site-isolation require a modification of the component being isolated that alters the efficacy of the reaction. The pot-in-pot approach, on the other hand, avoids many of these complications.

As mentioned above, “pot-in-pot” reactions are set up similar to *matryoshka* dolls (Russian nesting dolls) [30]. One of the primary advantages of this approach is that *unmodified* reaction systems can be site-isolated from one another, thereby forgoing the need for any alteration of the reaction conditions that may adversely affect the outcome of the reaction.

ILs played a key role in the design and deployment of pot-in-pot reactions based on an understanding of their role in selective flux as highlighted in Section 4 of this review [31]. Their properties make them uniquely suited to complement the site-isolation achieved by hydrophobic polymer membranes, particularly polydimethylsiloxane (PDMS). Since ILs cannot pass through a PDMS membrane [43,44,45,46], they are one of the few solvents that both remain site-isolated and are aprotic. Taking advantage of these attributes, ILs were used as a co-solvent for olefin metathesis, catalyzed by Grubbs second-generation catalyst, inside a PDMS thimble.

### 4.1. Site-Isolation of Incompatible Reactions

**Figure 4:**
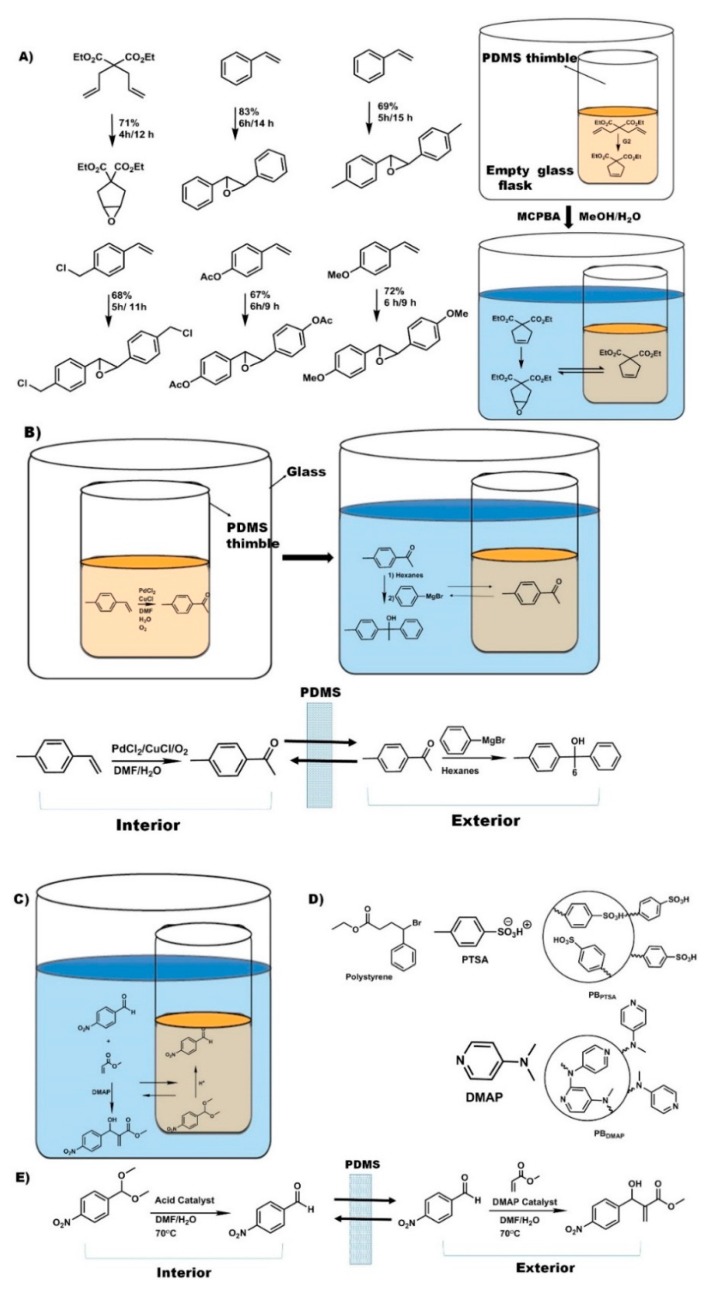
Examples of pot-in-pot reactions in which ionic liquids are employed as co-solvent (**A**). Alternatively, polar solvents (**B**) or reagent immobilization (**C**) is needed to avoid catalysts or reagent leakage through the polymer membrane. Polymer bound acid catalyst (**D**) and its use in a pot-in-pot reaction sequence (**E**).

In organic synthesis, strong nucleophiles and electrophiles (e.g., acids and bases) are the most widely known incompatible reagents, since they spontaneously react on mixing, often exothermically and with catastrophic results if not well managed. A common example of this is the use of nucleophilic group I and II organometallics compounds. Pot-in-pot reactions, however, have the ability to upend these limitations and make such reactions not only possible, but also easy (Figure 4). In one of the first examples of cascade reactions using a pot-in-pot approach, LiAlH_4_ and Grignard reagents were used in the presence of water [47]. A cyclic ketal was deprotected in aqueous conditions inside a PDMS (or polycyclooctene/polydicyclopentadiene) thimble, and then the ketone product of that reaction diffused through the PDMS membrane to be reduced to alcohol by LiAlH_4_ in an organic solvent on the exterior of the PDMS thimble.

Site-isolation of organometallic catalysts has long been the subject of research, and many different approaches have been developed. Catalysts may be bound to polymeric supports [48], heterogeneous surfaces [49], or other materials [50,51], or may be encapsulated in micelles [52,53,54], sol-gels [55,56,57,58], or zeolites [59,60,61,62,63,64,65,66,67,68]. Many of these approaches, however, require modification of the catalyst or reaction conditions in order to be successful. In contrast, the pot-in-pot technique can be carried out with little or no modification of the reaction conditions, since the reaction vessel, rather than the reaction itself, is modified (Figure 4).

The first report of site-isolating organometallic catalysts by the pot-in-pot approach detailed the use of Grubbs catalyst site-isolated from a strong oxidizer (*m*-CPBA) by a PDMS thimble (Figure 4a) [31]. This procedure also included the use of a room-temperature ionic liquid (1-butyl-3-methylimidazolium hexafluorophosphate ([BMIM PF_6_])) as a co-solvent on the interior of the thimble. Since it was insoluble in PDMS, it would neither diffuse out to the exterior solution nor swell the membrane. Since CH_2_Cl_2_ swells PDMS (thereby promoting flux through the membrane) while [BMIM PF_6_] does not, the two solvents could be used in combination on the interior of the PDMS thimble to tune the permeability of the membrane by adjusting the ratio of the two co-solvents.

Optimizing the permeability of the membrane is paramount to achieving selective diffusion, *i.e.*, to enabling the organic substrates to diffuse to the exterior solution while simultaneously preventing catalyst leaching. In this work, 1:1 ratio of CH_2_Cl_2_:[BMIM PF_6_] was optimal for site-isolating the catalyst while simultaneously facilitating the reaction cascade. By employing this solvent combination, 99.5% of the Ru catalyst remained on the interior of the PDMS thimble while allowing complete conversion.

### 4.2. Pot-in-Pot Reactions for Difficult to Handle Reagents and as Solution to Simple But Tedious Synthesis

**Figure 5:**
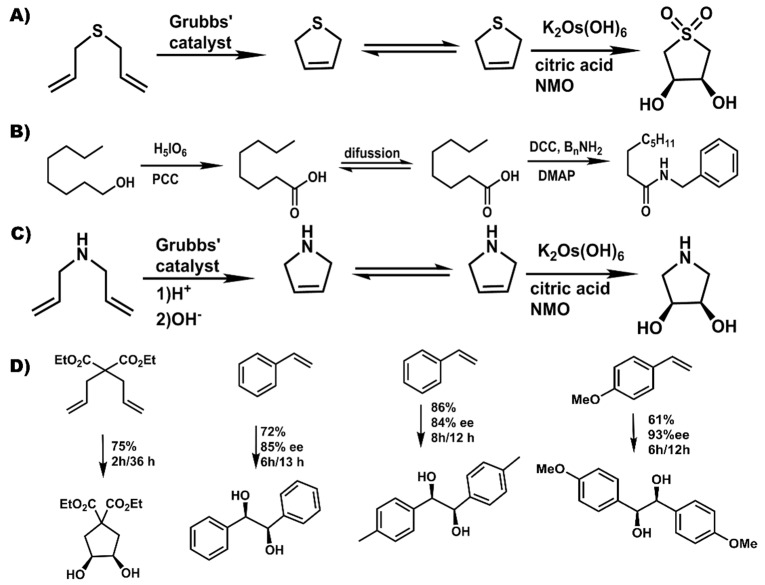
Examples of challenging or asymmetric transformations achieved using pot-in-pot reactions. (**A**) diallysulfide is converted to 1,1-dioxio-3,4-thiopheneoxide; (**B**) an alcohol is converted to an amide; (**C**) amine is converted to 2,5-dihydropyrrole; and (**D**) asymmetric transformation of metathesis products with high enantioselectivity or diastereoselctivity.

Besides adaptation of well-known reaction transformations, use of pot-in-pot to convert volatile compounds (Figure 5a) into solid and easy to handle final products has been reported [69]. The high solubility of the diallyl sulfide reagents in the ionic liquid mixture allows them to remain dissolved and hence be transformed to an even more volatile product, the 2,5-dihydrothiophene. Due to a more favorable partition coefficient into the PDMS membrane, the dihydrothiophene readily flux through the membrane, and is oxidized both at the thiolate and across the olefin to give *cis*-1,1-dioxio-3,4-thiopheneoxide in high yields and stereoselectivity. Besides reagents, some catalysts are also volatile, toxic, and, difficult to handle or re-use. The osmium based Sharpless dihydroxylation catalysts are such examples. The active species is the highly toxic OsO_4_ that is often generated from pre-catalysts like K_2_Os(OH)_6_, hence the need to regenerate them *in situ*. In such a case there is need for multi-phase reaction systems and a desire to recycle such catalysts. The use of ionic liquids in the first pot allows such a system to be established and maintained over long reaction times since they do not leach, and hence do not interfere with the biphasic medium needed for this regeneration.

Besides volatile reagents, there are reagents that require simple, well established transformations to make them compatible with a catalyst or reaction transformation. The use of amines with Grubbs catalysts is such an example. Protonation of the amine to mitigate chelation with the catalysts allows olefin metathesis, after which deprotonation (by altering the pH) regenerates the naked amine. Besides allowing compatibility, protonation of such reagents also makes them more soluble in the ionic liquid based medium, hence reducing partition into the polymer matrix and allowing higher conversion of the diallyl amine into the dihydropyrolium adduct (Figure 5c). Deprotonation and flux across the membrane followed by asymmetric sharpless dihydroxylation gives *cis*-3,4-pyrrolidinediol in 80% yield and 100% stereoselectivity. Analogously, a Mo’ Hunsen oxidation [70] of an alcohol followed by amidation leads to a transformation similar to that achieved by the Milstein’s catalyst [71], but, in a faster and more affordable non-catalytically process.

Other asymmetric hydroxylations that have been reported [69] included exploitation of the Thorpe-Ingold (*gem*-dimethyl) effect for high rates in the first reaction—hence limited partition of the starting reagent into the polymer, followed by dihydroxylation or use of styrenic moieties that can auto-polymerize in the polymer membrane in their unreacted form leading to much slower flux (Figure 5d). In the either case, the reactants had a significant dwell time in the ionic liquid medium allowing for their transformation into the olefin metathesis products that then flux into the second reaction medium where they are dihydroxylated in good yields and steroselectivities (Figure 5c).

## 5. Conclusions

In this review, we highlight the often undiscussed role of ionic liquids in the successful design of pot-in-pot reactions and offer insight to how understanding the fundamentals of mass transport across polymeric membranes can allow one to design reaction systems that circumvent handling of toxic reagents, cut down reaction times and eliminate purification steps. Introduction of ionic liquids as a major solvent while also deploying them as a delivery vehicle for a small amount of solvent—that eventually partitions evenly into the polymer membranes upon contact and swells/solvates the chains to increase flux-is critical for successful and efficient pot-in-pot reactions. The use of polymer membranes in selective flux of target molecules is highlighted through various classes of organic transformations.
